# Structural basis for SdgB- and SdgA-mediated glycosylation of staphylococcal adhesive proteins

**DOI:** 10.1107/S2059798321010068

**Published:** 2021-10-20

**Authors:** Dong-Gyun Kim, Inwha Baek, Yeon Lee, Hyerry Kim, Jin Young Kim, Geul Bang, Sunghwan Kim, Hye Jin Yoon, Byung Woo Han, Se Won Suh, Hyoun Sook Kim

**Affiliations:** a Research Institute, National Cancer Center, Goyang, Gyeonggi 10408, Republic of Korea; b Research Institute of Pharmaceutical Sciences, College of Pharmacy, Seoul National University, Seoul 08826, Republic of Korea; c Department of Chemistry, College of Natural Sciences, Seoul National University, Seoul 08826, Republic of Korea; d Department of Biological Chemistry and Molecular Pharmacology, Harvard Medical School, Boston, MA 02115, USA; e R&D Center, Voronoi Inc., Incheon 21984, Republic of Korea; f Korea Basic Science Institute, Ochang, Chungbuk 28119, Republic of Korea

**Keywords:** glycosyltransferases, SdgB, SdgA, serine–aspartate repeats, crystal structure, *Staphylococcus aureus*, staphylococcal adhesion

## Abstract

The initiation of infection of host tissues by *Staphylococcus aureus* requires a family of staphylococcal adhesive proteins containing serine–aspartate repeat (SDR) domains, such as ClfA. The O-linked glycosylation of the long-chain SDR domain mediated by SdgB and SdgA is a key virulence factor that protects the adhesive SDR proteins against host proteolytic attack in order to promote successful tissue colonization, and has also been implicated in staphylococcal agglutination, which leads to sepsis and an immunodominant epitope for a strong antibody response. Despite the biological significance of these two glycosyltransferases involved in pathogenicity and avoidance of the host innate immune response, their structures and the molecular basis of their activity have not been investigated. This study reports the crystal structures of SdgB and SdgA from *S. aureus* as well as multiple structures of SdgB in complex with its substrates (for example UDP, *N*-acetylglucosamine or SDR peptides), products (glycosylated SDR peptides) or phosphate ions. Together with biophysical and biochemical analyses, this structural work uncovered the novel mechanism by which SdgB and SdgA carry out the glycosyl-transfer process to the long SDR region in SDR proteins. SdgB undergoes dynamic changes in its structure such as a transition from an open to a closed conformation upon ligand binding and takes diverse forms, both as a homodimer and as a heterodimer with SdgA. Overall, these findings not only elucidate the putative role of the three domains of SdgB in recognizing donor and acceptor substrates, but also provide new mechanistic insights into glycosylation of the SDR domain, which can serve as a starting point for the development of antibacterial drugs against staphylococcal infections.

## Abstract 2

The crystal structures of SdgB and SdgA from *Staphylococcus aureus* provide functional and structural insights into the glycosylation mechanism in staphylococcal adhesion.

## 1. Introduction   

*Staphylococcus aureus* has long been recognized as a major human pathogen which underlies a wide spectrum of infections, ranging from skin and soft-tissue infections such as abscesses, furuncles and cellulitis to serious life-threatening conditions such as bloodstream infection, sepsis, pneumonia, endocarditis, and bone and joint infections (Liu, 2009 ▸; Archer, 1998 ▸). The emergence of *S. aureus* that is resistant to antibiotics, such as methicillin-resistant *S. aureus* (MRSA), poses a formidable therapeutic challenge (Lee *et al.*, 2018 ▸; Knox *et al.*, 2015 ▸). In fact, MRSA has become a leading cause of bacterial infections in both healthcare and community settings owing to its capacity for genetic adaption (Turner *et al.*, 2019 ▸). The high mortality and morbidity rates associated with MRSA infection highlight the need for alternative therapeutic agents targeting MRSA.

The adhesion of *S. aureus* to the extracellular matrix or to the surface of host cells is a prerequisite for tissue colonization and initiation of infection. *S. aureus* surface proteins including serine–aspartate repeats (SDR proteins) play an important role in tissue colonization (Foster & Höök, 1998 ▸). Clumping factor A (ClfA) is the most extensively studied SDR protein and is known to be involved in triggering sepsis (Flick *et al.*, 2013 ▸; McAdow *et al.*, 2011 ▸; Higgins *et al.*, 2006 ▸; Loof *et al.*, 2015 ▸). ClfB, SdrC, SdrD and SdrE are other members of the SDR proteins (Cheng *et al.*, 2012 ▸; McCrea *et al.*, 2000 ▸). The SDR proteins contain an N-terminal ligand binding A domain, an SDR domain and a C-terminal LP*X*TG motif (Clarke & Foster, 2006 ▸; Cheng *et al.*, 2012 ▸). The SDR domain contains 25–275 SD repeats, which can be heavily glycosylated, and the glycosylated SD repeats act as a mechanical barrier contributing to the evasion of host defenses (Thomer *et al.*, 2014 ▸; Hazenbos *et al.*, 2013 ▸). Sugar moieties on the SDR domains can also promote abscess formation, allowing the bacteria to reside and disseminate without being attacked by the host immune system (Vernachio *et al.*, 2003 ▸; Cheng *et al.*, 2010 ▸).

Two glycosyltransferases (GTases), SdgB and SdgA, are responsible for the glycosylation of *S. aureus* SDR proteins such as ClfA and ClfB. SdgB first appends *N*-acetyl­glucosamine (GlcNAc) moieties onto serine residues (O-glycosylation) within the SDR domain using uridine diphos­phate *N*-acetylglucosamine (UDP-GlcNAc) as a donor substrate (Hazenbos *et al.*, 2013 ▸). The subsequent addition of GlcNAc to the glycoproteins is catalyzed by the second enzyme, SdgA, yielding glycosylated SDR proteins in which each SD repeat is decorated with GlcNAc disaccharide moieties (Hazenbos *et al.*, 2013 ▸). The genes for SdgA and SdgB, which are highly conserved in all sequenced *S. aureus* genomes, are found directly adjacent to the genes encoding a subset of their targets: SdrC, SdrD and SdrE (Hazenbos *et al.*, 2013 ▸). The glycosylation of SDR proteins mediated by SdgA and SdgB protects SDR proteins from degradation by host proteases, thereby circumventing innate immune attack. The GlcNAc modification of SDR proteins by SdgB has also been implicated in staphylococcal agglutination in human plasma, which leads to sepsis, although it also elicits an immuno­dominant epitope for a strong antibody response (Thomer *et al.*, 2014 ▸; Hazenbos *et al.*, 2013 ▸). Despite the biological significance of the sugar modification of the SDR domain by SdgB and SdgA, the structural and molecular basis of SdgB and SdgA remains poorly understood.

This study aimed to determine the crystal structures of SdgB and SdgA as well as the structures of SdgB in complex with its donor (UDP-GlcNAc) and acceptor (SD peptide) substrates to understand the *O*-GlcNAc glycosylation of SDR domains by SdgB and SdgA at the molecular level. We have characterized biochemical, biophysical and structural features of SdgB and SdgA from *S. aureus* USA300, which is the most virulent clinical strain of MRSA. We found that SdgB and SdgA have a unique inserted domain, which is used to form a homodimer or heterodimer of SdgB and SdgA. In addition to the dimerization role, the inserted domain was found to serve as a binding platform for the SD repeats before and after glycosylation. A long positive tract along the inserted domain stabilizes the binding of the glycosylated SD repeats to SdgB by offsetting the negative charge clustered in the tandem SD repeats. Interestingly, SdgB undergoes a conformational change from an open to closed complex upon substrate binding. SdgB and SdgA share the way that they recognize monoglycosylated SD repeats as a product and a substrate, respectively, but SdgA is likely to preferentially bind a longer SDR substrate than a shorter one. Altogether, the extensive snapshots of SdgB complexes in multiple states provided a mechanistic insight into how SdgB recognizes and glycosylates the clustered serine residues in the SDR proteins. We hope that the structural information described here will serve as a foundation for a novel strategy for the development of a therapeutic agent against staphylococcal infections.

## 2. Materials and methods   

### 2.1. Gene cloning, protein expression and purification   

The genes *SAUSA300_0550* and *SAUSA300_0549* encoding SdgB and SdgA, respectively, were amplified using PCR and cloned into pET-21a(+) (Novagen, Burlington, Massachusetts, USA) to fuse a His_6_ tag at the C-terminus. The recombinant SdgB and SdgA proteins were overexpressed in *Escherichia coli* Rosetta 2(DE3)pLysS cells (Sigma–Aldrich, St Louis, Missouri, USA). The cells were grown at 25 or 20°C for SdgB and SdgA, respectively, in Luria–Bertani (LB) broth. For selenomethionine (SeMet)-substituted proteins, M9 minimal medium was used. When the cells reached an OD_600_ of 0.8–1.0, 0.5 m*M* isopropyl β-d-1-thiogalactopyranoside (IPTG) was added to induce protein overexpression, followed by further incubation for 16 h. In the case of SeMet-substituted protein cultures, 50 mg l^−1^ SeMet, 100  mg l^−1^ phenylalanine, 100 mg l^−1^ threonine, 100 mg l^−1^ lysine, 50 mg l^−1^ leucine, 50 mg l^−1^ isoleucine, 50 mg l^−1^ valine and 50 mg l^−1^ proline were added to the cells 30 min before induction. The cells were then transferred to a 15°C incubator, grown for an additional 20 h and harvested by centrifugation at 4300*g* for 10 min. The harvested cells were lysed using a cell sonicator (SONICS) in a lysis buffer consisting of 20 m*M* Tris–HCl pH 7.5, 500 m*M* NaCl, 35 m*M* imidazole, 10%(*v*/*v*) glycerol, 1 m*M* phenylmethylsulfonyl fluoride. The lysate was centrifuged at 35 000*g* for 60 min at 4°C and the filtered supernatant was loaded onto a HiTrap Chelating HP column (GE Healthcare, Chicago, Illinois, USA) pre-equilibrated with lysis buffer. The SdgB and SdgA proteins eluted in imidazole concentration ranges of 60–400 and 100–250 m*M*, respectively. The eluted fractions were applied onto a HiTrap Q column (GE Healthcare) equilibrated with a buffer consisting of 20 m*M* Tris–HCl pH 9.0, 75 m*M* NaCl and were eluted with a linear gradient of NaCl from 75 to 500 m*M*. The proteins were further purified by gel filtration on a HiLoad 16/600 Superdex 200 prep-grade column (GE Healthcare) pre-equilibrated with a buffer consisting of 20 m*M* Tris pH 7.9, 200 m*M* NaCl or a buffer consisting of 20 m*M* Tris pH 7.0, 200 m*M* NaCl for SdgB or SdgA, respectively. The final SdgB and SdgA proteins used for crystallization were prepared at 4.9 and 8.4 mg ml^−1^, respectively.

### 2.2. Crystallization and X-ray data collection   

Crystals of SdgB and SdgA were grown at 14°C by the sitting-drop vapor-diffusion method by mixing equal volumes of the protein and a crystallization solution. Crystals were grown in several conditions and were soaked with donor or acceptor substrates with various concentrations and incubation times: (i) 0.2 *M* MgCl_2_, 0.1 *M* Tris–HCl pH 8.5, 25%(*w*/*v*) polyethylene glycol (PEG) 3350 for the ligand-free SdgB crystal (SdgB_unbound_), the SdgB crystal quick-soaked with 6.14 m*M* UDP and 2.45 m*M* 5-mer SD-repeat peptide (DSDSD) for 30 min (SdgB_UDP–peptide_) and the SdgB crystal incubated with 2.45 m*M* 3-mer SD-repeat peptide and 6.14 m*M* UDP-GlcNAc, although only the peptide was visible in the structure (SdgB_peptide_), (ii) 0.2 *M* calcium acetate, 0.1 *M* MES pH 5.5, 20%(*w*/*v*) PEG 8000 for the ligand-free SdgA structure (SdgA_unbound_), (iii) 20 m*M* CaCl_2_, 85 m*M* trisodium citrate pH 5.6, 25.5%(*w*/*v*) PEG 4000, 15%(*w*/*v*) glycerol for the SdgB crystal soaked with 2.66 m*M* 9-mer SD-repeat peptide and 9.60 m*M* UDP-GlcNAc for 7.5 h, resulting in the diGlcNAcylated peptide–UDP–GlcNAc-bound form (SdgB_quaternary_), and (v) 1.2 *M* NaH_2_PO_4_, 0.8 *M* K_2_HPO_4_, 0.1 *M* CAPS–NaOH pH 10.5 for the phosphate-bound SdgB structure (SdgB_phosphate_). Crystals of diffraction quality were briefly immersed in reservoir solution containing an additional 20–25%(*v*/*v*) glycerol and were flash-cooled in liquid nitrogen. Due to the absence of a search model for the molecular-replacement method, SeMet-substituted crystals of SdgB were grown at 14°C to solve the phase problem. Diffraction data from the SeMet-substituted or native crystals were collected at 100 K to resolutions of 1.85–3.20 Å on beamlines PF-17A, PF-1A and NE-3A at Photon Factory, Japan (data sets 1, 4 and 7, respectively, in Table 1 ▸) and on beamlines PLS-7A (data sets 2, 5 and 6) and PLS-5C at Pohang Light Source, Korea (data set 3). Raw X-ray diffraction data were indexed and scaled using the *HKL*-2000 suite (Otwinowski & Minor, 1997 ▸); data-collection statistics are listed in Table 1 ▸.

### 2.3. Structure determination, refinement and analysis   

Single-wavelength anomalous diffraction (SAD) phases of SeMet-substituted SdgB were initially calculated with *AutoSol* from the *Phenix* software suite (Terwilliger *et al.*, 2009 ▸; Liebschner *et al.*, 2019 ▸) and were further improved by the automatic model-building program *RESOLVE* (Terwilliger, 2003 ▸), resulting in an initial model. The initial model was further refined to the final model using iterative cycles of model building with *Coot* (Emsley *et al.*, 2010 ▸) and subsequent refinement with *REFMAC*5 in the *CCP*4 suite (Murshudov *et al.*, 2011 ▸) and *phenix.refine* (Adams *et al.*, 2010 ▸). The crystal structures of native SdgB in ligand-bound and unbound forms and native SdgA were determined by molecular replacement (MR) with *MOLREP* (Vagin & Teplyakov, 2010 ▸), using the refined structure of SeMet-substituted SdgB as a phasing model. Validation of crystal structures was implemented with *MolProbity* (Chen *et al.*, 2010 ▸) and the Research Collaboratory for Structural Bioinformatics (RCSB) Protein Data Bank (PDB) validation server.

### 2.4. Size-exclusion chromatography with multi-angle light scattering   

The oligomeric states of the recombinant SdgB and SdgA proteins were assessed by SEC-MALS experiments using an ÄKTA fast protein liquid-chromatography (FPLC) system (GE Healthcare) connected to a Wyatt DAWN HELEOS II MALS instrument and a Wyatt Optilab T-rEX differential refractometer (Wyatt, Santa Barbara, California, USA). A Superdex 200 Increase 10/300 GL (GE Healthcare) gel-filtration column pre-equilibrated with a buffer consisting of 20 m*M* Tris–HCl pH 7.5, 200 m*M* NaCl or a buffer consisting of 20 m*M* Tris–HCl pH 8.5, 200 m*M* NaCl for SdgB or SdgA, respectively, was normalized using ovalbumin. The SdgB (0.22 mg) or SdgA (0.23 mg) proteins were injected at a flow rate of 0.5 ml min^−1^. The data were analyzed using the Zimm model for fitting static light-scattering data and graphs were otained using *EASI Graph* (Easy Analytic Software Inc.) with an ultraviolet (UV) peak in the *ASTRA *6 software (Wyatt).

### 2.5. Sedimentation-velocity and sedimentation-equilibrium analytical ultracentrifugation   

To determine the oligomeric state of recombinant SdgB and SdgA in solution, we performed sedimentation-equilibrium and sedimentation-velocity experiments using a ProteomeLab XL-A Analytical Ultracentrifuge (Beckman Coulter). Sedimentation-equilibrium analysis was performed on SdgB (1 µ*M*) and SdgA (4.5 µ*M*) prepared in 20 m*M* HEPES buffer pH 7.5 containing 150 m*M* sodium chloride and 1 m*M* MgCl_2_, and the same buffer was used as a blank. The protein concentrations of the recombinant SdgB and SdgA proteins were calculated using ɛ_280 nm_ = 59 772.8 and 58 547.5 *M* ^−1^ cm^−1^, respectively. Each sample was spun until equilibrium for 24 h at two speeds (9000 and 15 000 rev min^−1^), monitoring the absorbances of SdgB and SdgA at 230 or 280 nm. For the sedimentation-velocity experiment, SdgB (0.5, 1.0 and 4.5 µ*M*) and SdgA (0.375, 1.0 and 4.5 µ*M*) were spun in double-sector cells at 30 000 rev min^−1^. The sedimentation-equilibrium and sedimentation-velocity data sets were analyzed by *SEDFIT* and *SEDPHAT*, respectively (Zhao *et al.*, 2013 ▸). To measure the *K*_d_ value for dimerization, the sedimentation data set was globally fitted to a monomer–dimer self-association model.

### 2.6. *O*-GlcNAcyltransferase assay by mass analysis   

Synthetic serine–aspartate repeat (SDR) 3-mer and 5-mer peptides (DSD and DSDSD) were used as potential substrates for the *O*-GlcNAcyltransferase assay. 3 m*M* DSD or DSDSD was incubated for 2 h at 37°C with either recombinant SdgB, SdgA or both proteins at 5 µ*M* along with 10 m*M* UDP-GlcNAc in reaction buffer (20 m*M* HEPES pH 7.5, 150 m*M* NaCl, 1 m*M* MgCl_2_). A 1 µl aliquot of the reaction mixture was dropped onto the target plate and mixed with 2,5-di­hydrobenzoic acid (DHB) as a matrix. DHB was dissolved in 50:50(*v*:*v*) acetonitrile:water containing 0.5% trifluoroacetic acid (TFA) at a concentration of 10 mg ml^−1^. The MS spectra were obtained in a positive reflection mode (*R* = 15 000) using a Bruker UltrafleXtreme matrix-assisted laser desorption/ionization (MALDI) MS instrument (Bremen, Germany) equipped with a SmartBeam II laser. As a control, we detected the peaks corresponding to the 3-mer and 5-mer peptides (Figs. 2*a* and 2*b*), which were converted into mono-GlcNAcylated forms by SdgB but not by SdgA.

### 2.7. Surface plasmon resonance   

The kinetics and affinity of SdgB for SdgA were investigated by surface plasmon resonance (SPR) using a Reichert SR7500 dual-channel instrument (Reichert, Depew, New York, USA). The SdgB protein purified in 20 m*M* HEPES pH 7.5, 200 m*M* sodium chloride was immobilized on a PEG-based surface sensor chip (Reichert) at 20 µl min^−1^ to a 1062 RU immobilization level with HBS buffer (10 m*M* HEPES pH 7.4, 150 m*M* NaCl). The running buffer used for the interaction study of SdgB and SdgA was HBS-EP buffer consisting of 10 m*M* HEPES pH 7.4, 150 m*M* sodium chloride, 0.03 m*M* EDTA, 0.005%(*v*/*v*) Tween 20. All SPR experiments were performed at 20°C. SdgA samples at concentrations of 0.39, 0.78, 1.56, 3.13, 6.25, 12.5 and 25.0 µ*M* were prepared in HBS-EP buffer. Serially diluted analytes were injected over the SdgB chip at 30 min^−1^ for 3 and 10 min for association and dissociation analyses, respectively. Subsequently, regeneration of the chip was carried out using 10 m*M* sodium hydroxide for 30 s between cycles. The binding was detected as a change in the refractive index at the surface of the chip as measured in response units (RU). The kinetics SPR data were fitted using the *Scrubber*2 software (Wei & Latour, 2008 ▸).

### 2.8. Data availability   

The coordinates and structure factors have been deposited in the Protein Data Bank under the following accession codes: 7vfk for SdgB_unbound_ (ligand-free), 7ec2 for SdgA_unbound_ (ligand-free), 7vfl for SdgB_quaternary_ (SdgB–UDP–GlcNAc– diGlcNAcylated peptide complex), 7vfm for SdgB_UDP–peptide_ (SdgB–UDP–5-mer complex), 7vfn for SdgB_peptide_ (SdgB–3-mer complex) and 7vfo for SdgB_phosphate_ (SdgB–phosphate complex). Other data reported in this manuscript are available from the corresponding author upon reasonable request.

## 3. Results   

### 3.1. SdgB has *O*-GlcNAcylation activity on the minimum DSD motif of SDR   

**Figure 1:**
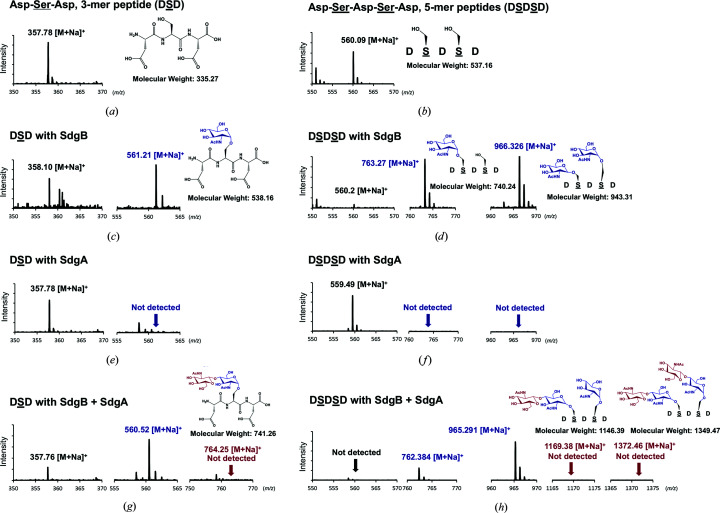
GTase activity of SdgB and SdgA using mass spectrometry. (*a*, *b*) Mass spectra of the synthesized DSD (*a*) and DSDSD (*b*) peptides. The observed peaks correspond to a sodium adduct [*M*+Na]^+^ of the peptides. (*c*, *d*) Mass spectra of the DSD (*c*) and DSDSD (*d*) peptides incubated with SdgB and UDP-GlcNAc. The expected *O*-GlcNAcylation of serine is shown as a blue chemical structure to the right of the peak. (*e*, *f*) Mass spectra of the peptides incubated with SdgA and UDP-GlcNAc. GlcNAcylation was not detected. (*g*, *h*) Mass spectra of the peptides incubated with both SdgB and SdgA in the presence of UDP-GlcNAc. The expected second GlcNAcylations by SdgA, which are shown as red chemical structures to the right of the peak, were not detected in this study. All mass values are listed as the monoisotopic mass.

SdgB and SdgA have been reported to append GlcNAc moieties to SDR domains in a sequential manner, in which the SDR targets are first modified by SdgB, followed by further modification by SdgA (Hazenbos *et al.*, 2013 ▸). To verify the molecular function of SdgB and SdgA in O-linked glycosyl­ation of SD repeats *in vitro*, the glycosyltransferase activity of SdgB and SdgA was measured using mass analysis with UDP-GlcNAc and synthetic SDR peptides [Asp-Ser-Asp (DSD) and Asp-Ser-Asp-Ser-Asp (DSDSD)] as potential substrates. DSD and DSDSD peptides were detected at about *m*/*z* = 358 and 560, respectively, as the charged species bound to a sodium ion ([*M*+Na]^+^; Figs. 1 ▸ *a* and 1 ▸ *b*). When the peptides were incubated with recombinant SdgB protein, the *O*-GlcNAcylated products mono-GlcNAcylated DSD (Fig. 1 ▸ *c*) and mono- or di-GlcNAcylated DSDSD (Fig. 1 ▸ *d*) were detected, which validates the ability of SdgB to recognize and modify the minimum DSD motif. In contrast, incubation of the DSD or DSDSD peptides with SdgA did not show any modified products in the absence of SdgB (Figs. 1 ▸ *e* and 1 ▸ *f*). SdgA alone was not able to glycosylate the SD peptides, which is consistent with previous findings, in which GlcNAc glycosyl­ation of SDR targets by SdgB was required for the GTase activity of SdgA (Hazenbos *et al.*, 2013 ▸; Thomer *et al.*, 2014 ▸). However, sequential or simultaneous incubation of SdgB and SdgA with 3-mer and 5-mer SDR peptides still yielded only the first products (mono-GlcNAcylated DSD and mono- or di-GlcNAcylated DSDSD) modified by SdgB (Figs. 1 ▸ *g* and 1 ▸ *h*). The second products (di-GlcNAcylated DSD and tri- or tetra-GlcNAcylated DSDSD) that were expected to be additionally modified by SdgA were not observed (Figs. 1 ▸ *g* and 1 ▸ *h*), suggesting that 3-mer and 5-mer SDR peptides are too short to be modified by SdgA. Taken together, SdgB and SdgA exhibited a difference in explicit glycosyltransferase activity towards the minimum DSD motif of SDR despite their high sequence similarity.

### 3.2. The overall structures of SdgB and SdgA share the GT-B fold with a unique inserted domain   

**Figure 2:**
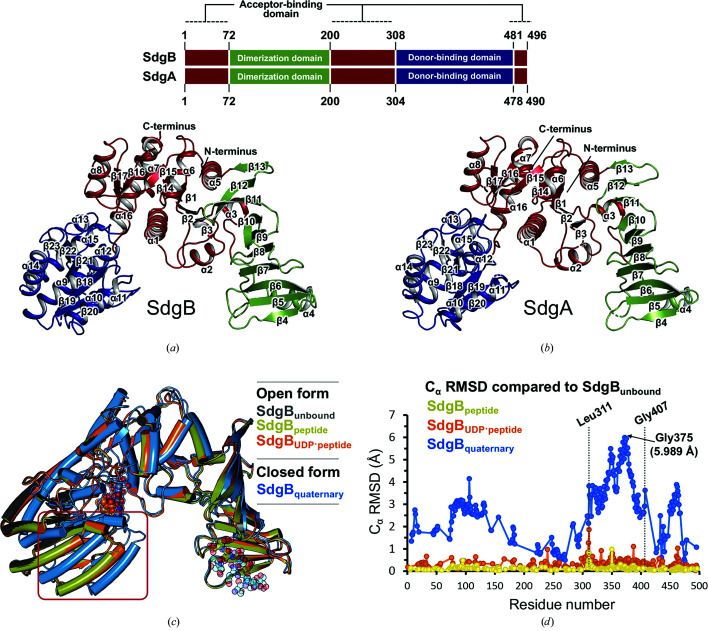
Crystal structures of SdgB_unbound_ and SdgA_unbound_. (*a*, *b*) The monomeric structures of SdgB (*a*) and SdgA (*b*) are presented as ribbon diagrams. Each monomer consists of three domains: the acceptor (SDR)-binding domain (ABD), the dimerization domain and the donor (UDP-GlcNAc)-binding domain (DBD), which are distinguished in red, green and blue, respectively. Top: schematic of the SdgB and SdgA sequences showing the domain composition. (*c*) A superimposed view of the SdgB structures. The *A* chains of the SdgB structures are presented as cylindrical helices, which were overlaid using *SSM* in *Coot*. (*d*) C^α^ r.m.s.d. plot of SdgB_peptide_, SdgB_UDP–peptide_ and SdgB_quaternary_ structures compared with SdgB_unbound_. The r.m.s.d. per residue was calculated by *LSQKAB* in *CCP*4 using the *A* chains of the SdgB structures. In (*c*) and (*d*), the red box and the black dotted lines indicate the most deviating region in the compared structures.

To examine the structural and mechanistic basis of SdgB function and to elucidate how it differs from SdgA in structure, we determined crystal structures of full-length SdgB and SdgA from *S. aureus* (strain USA300). The initial structure of SdgB, in which methionines were substituted with selenomethionines (SdgB_SeMet_), was solved at 2.80 Å resolution using the SAD method. Next, the native crystal structure of SdgB (SdgB_unbound_; PDB entry 7vfk; Fig. 2 ▸ *a*) was determined at the high resolution of 1.85 Å by molecular replacement (MR) using SdgB_SeMet_ as a search model. The structure of SdgA (SdgA_unbound_; PDB entry 7ec2; Fig. 2 ▸ *b*) was solved at a resolution of 2.4 Å by MR using SdgB_unbound_ as a search model. In addition, structures of SdgB complexed with diverse ligands including UDP, UDP-GlcNAc, SD-repeat peptides, glycosylated SD-repeat peptides and phosphate ions were subsequently determined in the resolution range 1.9–3.2 Å; the overall structures of the glycosylated peptide–UDP–GlcNAc-bound form (SdgB_quaternary_), the peptide–UDP-bound form (SdgB_UDP–peptide_), the peptide-bound form (SdgB_peptide_) and the phosphate-bound form (SdgB_phosphate_) are structurally similar to each other, with root-mean-square deviations (r.m.s.d.s) of 2.28 Å (Figs. 2 ▸ *c* and 2 ▸ *d*), as discussed later. The crystallographic statistics of all data sets are shown in Table 1 ▸. We focus our analysis below on the highest-resolution SdgB_unbound_ structure, unless otherwise noted.

The structures of SdgB and SdgA, which share 44% sequence identity, reveal a high structural similarity, with an r.m.s.d. of 1.52 Å for the C^α^ positions of 467 aligned residues. Their overall structures showed an open, V-shaped form consisting of the catalytic domain and an inserted β-stranded domain (Figs. 2 ▸ *a* and 2 ▸ *b* and Supplementary Fig. S1). The catalytic domain of SdgB and SdgA possesses a canonical GT-B fold, which is commonly found in many other glycosyltransferases (Lairson *et al.*, 2008 ▸; Bourne & Henrissat, 2001 ▸; Janetzko & Walker, 2014 ▸). This fold is characterized by two separate Rossmann-like domains (β/α/β; Lairson *et al.*, 2008 ▸): one containing a donor-binding site (Figs. 2 ▸ *a* and 2 ▸ *b* and Supplementary Fig. S1, blue) and the other containing an acceptor-binding site (Figs. 2 ▸ *a* and 2 ▸ *b* and Supplementary Fig. S1, red). In SdgB, UDP-GlcNAc (the donor substrate) and the SDR region (the acceptor substrate) are expected to be bound to each domain. In addition to the Rossmann-like domains, SdgB and SdgA harbor a unique inserted domain (called the DUF1975 domain) consisting of ten antiparallel β-strands (Figs. 2 ▸ *a* and 2 ▸ *b* and Supplementary Fig. S1, green).

As analyzed by the *DALI* structural similarity search algorithm (Holm, 2020 ▸), the monomeric structures of SdgB and SdgA containing the inserted domain are similar to those of TarM, a teichoic acid α-glycosyltransferase from *S. aureus* (Sobhanifar *et al.*, 2015 ▸; PDB entry 4x6l; *Z*-scores of 33.5 and 34.1 and sequence identities of 21% and 22% to SdgB and SdgA, respectively), and the GtfA/B glycosyltransferase from *Streptococcus gordonii* (Chen *et al.*, 2016 ▸; PDB entry 5e9t; *Z*-scores of 35.1 and 33.4 and sequence identities of 21% and 23% to SdgB and SdgA, respectively), where the inserted domains contribute to oligomer assembly. However, the DUF1975 domain in each protein leads to different oligomeric states. The DUF1975 domain of TarM forms a trimeric structure, while that of GtfA and GtfB forms a heterodimeric interface between the two enzymes (Supplementary Figs. S2*b* and S2*c* ).

### 3.3. SdgB and SdgA can form homodimers and heterodimers   

**Figure 3:**
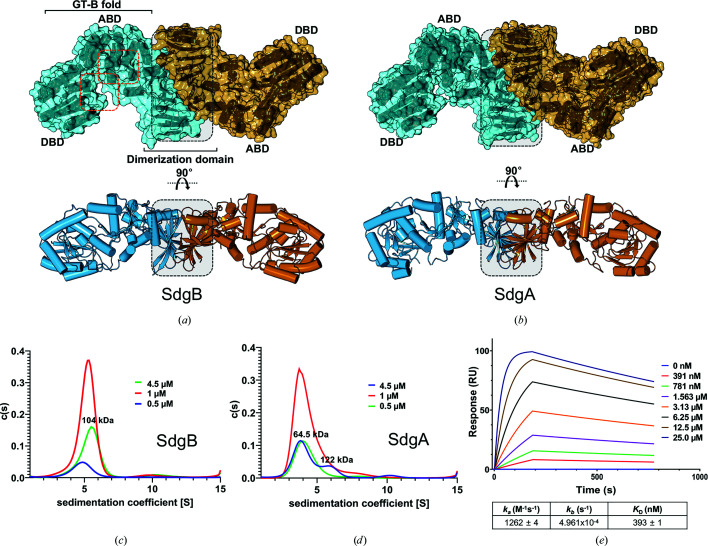
Dimeric structures of SdgB and SdgA. (*a*, *b*) The dimeric structures of SdgB (*a*) and SdgA (*b*) are presented as cylindrical models in surface view, and each monomer is colored sky and orange. The core domains are labeled, and the expected key sites and dimerization area are marked with orange and gray dotted lines, respectively. (*c*, *d*) The distribution of the sedimentation coefficient of SdgB (*c*) and SdgA (*d*) [*c*(*s*) versus *s*, where *s* is in svedbergs (S)] from the sedimentation-velocity experiments. (*e*) SPR analyses for measurement of the binding affinity between SdgB and SdgA. The SPR sensorgram shows the direct binding of SdgA (0.391–25.0 µ*M*) to immobilized SdgB. The calculated *k*_a_, *k*_b_ and *K*_d_ values are also shown below the plot.

In all SdgB and SdgA structures the crystallographic asymmetric unit contained two SdgB or SdgA molecules (chains *A* and *B*). *Proteins, Interfaces, Structures and Assemblies* (*PISA*) analysis (Krissinel & Henrick, 2007 ▸) showed that the largest interface area that each SdgB or SdgA molecule shares with an adjacent molecule in the crystals is 1285 Å^2^ (8.5% of the whole surface area of the monomer) or 1436 Å^2^ (9.5%), respectively, suggesting that both SdgB and SdgA can form dimers. The crystal structures of SdgB and SdgA also showed that the inserted domain appears to contribute to unique homodimeric interactions (Figs. 3 ▸ *a* and 3 ▸ *b* and Supplementary Fig. S2*a* ). Despite the *PISA* prediction showing that SdgA has a larger interface area than SdgB, the dimeric interface of SdgB reveals more hydrophobic and hydrophilic interactions than that of SdgA (Supplementary Figs. S3*a* and S3*b* ). In particular, the interface of SdgB possesses four salt bridges, Glu173_chain *A* or *B*_–Lys136_chain *B* or *A*_ and Glu204_chain *A* or *B*_–Arg107_chain *B* or *A*_, which are the combination of a hydrogen bond and a strong ionic bond. In contrast, the dimeric interface of SdgA does not show any salt bridges (Supplementary Fig. S3*c* ), suggesting that SdgB can form a more stable dimeric conformation than SdgA. To determine the oligomeric states of SdgB and SdgA in solution, we utilized size-exclusion chromatography with multi-angle light scattering (SEC-MALS). SdgB in solution eluted at a volume corresponding to a molecular weight of about 119 kDa (Supplementary Fig. S2*d* ), which is twice as large as the theoretical monomer mass of SdgB (59.5 kDa), suggesting that SdgB forms a homodimer. However, at the same concentration SdgA eluted at a volume corresponding to 60 kDa, which matches the theoretical monomer mass of SdgA (60.4 kDa; Supplementary Fig. S2*d* ), suggesting that SdgA exists as a monomer in solution. To explain the discrepancy between the SEC-MALS and structural data for SdgA, we examined the concentration-dependence of the oligomeric state using sedimentation-velocity analytical ultracentrifugation (SV-AUC). Three different concentrations of both SdgB and SdgA were tested (Figs. 3 ▸ *c* and 3 ▸ *d*). The SV-AUC data showed that SdgB exclusively exists as a single species, a dimer of 104 kDa, in the concentration range 0.5–4.5 µ*M*, while SdgA is present in the form of a mixture of a monomer (64.5 kDa) and a dimer (122 kDa) at concentrations as high as 4.5 µ*M*, while acting like a monomer (64.5 kDa) at a low concentration range. Given that their dimerization may be concentration-dependent, we additionally measured the binding affinity (*K*_d_) between two monomer molecules of SdgB or SdgA by sedimentation-equilibrium (SE) analytical ultracentrifugation, giving *K*_d_ values of 926 n*M* and 14.1 µ*M* for SdgB (Supplementary Fig. S2*e* ) and SdgA (Supplementary Fig. S2*f* ), respectively. These results reveal that SdgB and SdgA exist in a monomer–dimer equilibrium, but the interface analysis of the SdgB and SdgA structures suggests that SdgB has a much stronger tendency to achieve the dimeric form than SdgA.

Since SdgB and SdgA are structurally similar, and functionally and genomically associated, we examined whether SdgB and SdgA can form a heterodimer through their inserted domains. Interestingly, when measured using surface plasmon resonance (SPR), as shown in Fig. 3 ▸(*e*), SdgB could bind SdgA with a stronger affinity of 393 n*M* than in homodimers of SdgB or SdgA alone. By superimposing their dimeric structures, a model structure of the SdgB–SdgA heterodimer was presented (Supplementary Fig. S2*g* ), showing favored intermolecular interactions between dimerization domains in the SdgB–SdgA heterodimer. These results show the possibility that SdgB and SdgA could also form an SdgB–SdgA heterodimer in addition to SdgB or SdgA homodimers, presumably to efficiently append GlcNAc moieties on the same substrates in an ordered fashion.

### 3.4. Structure of SdgB in complex with UDP–GlcNAc and the *O*-GlcNAcylated SDR peptide   

**Figure 4:**
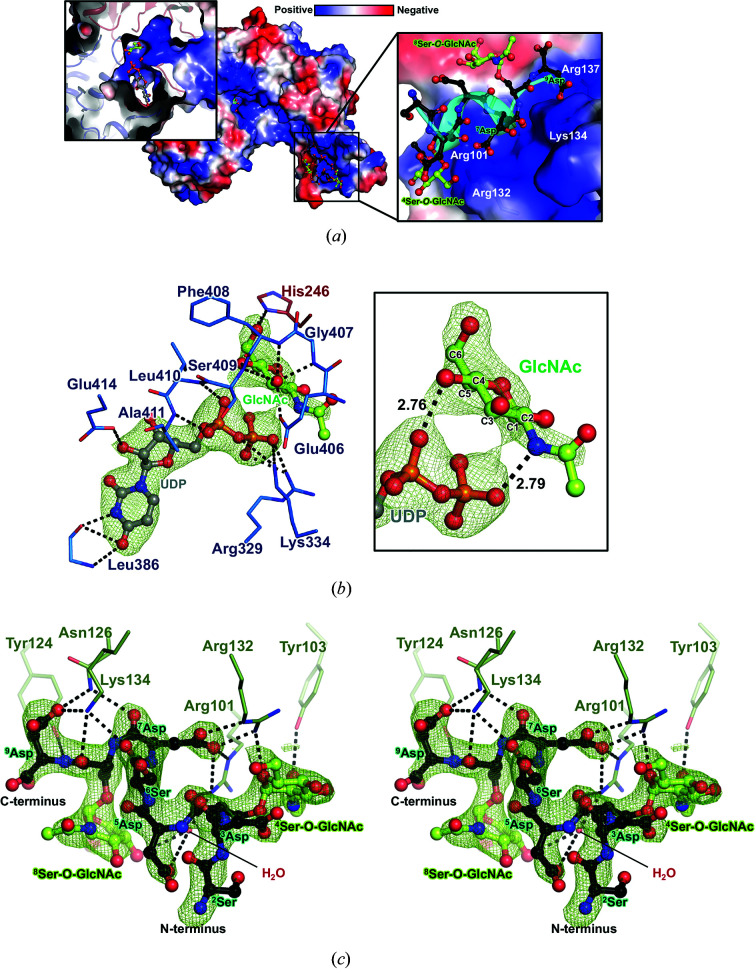
The quaternary complex of SdgB (SdgB_quaternary_) with UDP, GlcNAc and GlcNAcylated 9-mer SD-repeat peptide. (*a*) The electrostatic potential surface view of SdgB_quaternary_. UDP and the cleaved GlcNAc were observed in the cleft between the DBD and ABD, and the buried region was visualized in an enlarged view (left). The GlcNAcylated SD peptide attached inside the dimerization domain is shown as a stick and cartoon model (right). (*b*, *c*) Detailed binding modes of UDP and GlcNAc in the donor-binding site (*b*) and the GlcNAcylated product in the dimerization domain shown as a stereoview (*c*). The ligands are shown in ball-and-stick representation. The key residues for ligand binding are presented as lines with labels and the key interactions are shown as black dotted lines. The left panel in (*b*) highlights the interactions between UDP and GlcNAc, and the C atoms of GlcNAc are numbered. In (*b*) and (*c*), *mF*_o_ − *DF*_c_ omit maps of ligands are contoured at 3.0σ as green mesh. The C atoms of UDP, GlcNAc and the SDR peptide are colored gray, light green and black, respectively. O atoms, N atoms and phosphates are colored red, blue and orange, respectively.

To gain further mechanistic insight into how SdgB recognizes its substrates and stabilizes its products, we determined diverse complex structures of SdgB (Table 1 ▸). Interestingly, the quaternary-complex structure of SdgB (SdgB_quaternary_; PDB entry 7vfl) spontaneously shows a unique binding mode of UDP–GlcNAc as well as the *O*-GlcNAcylated SDR peptide (9-mer) to SdgB at a resolution of 2.45 Å (Fig. 4 ▸ *a*).

On detailed inspection of the active site, distinct electron density for UDP and GlcNAc was observed in the cleft formed between the donor-binding domain (DBD) and the acceptor-binding domain (ABD), indicating the cleavage of UDP-GlcNAc (Fig. 4 ▸ *b*). The UDP moiety is tucked into the shallow pocket formed by the DBD and the α1–α2 loop of the ABD (Figs. 4 ▸ *a* and 4 ▸ *b*). The uridine unit of UDP forms hydrogen bonds to the backbone amide and carbonyl O atom of Leu386, which is stabilized by hydrophobic interaction with Tyr358 and Leu389 and π–π stacking with Phe386 (Fig. 4 ▸ *b* and Supplementary Fig. S4*a* ). The ribose ring and the following α-phosphate make hydrogen bonds to Glu414 and the backbone amides of Leu410 and Ala411, as well as hydrophobic interactions with Gly15, Arg329 and Ser409 (Fig. 4 ▸ *b* and Supplementary Fig. S4*a* ). After the cleavage of UDP-GlcNAc, the β-phosphate of UDP is stabilized by the positive charge of Arg329 and Lys334 (Fig. 4 ▸ *b*). The GlcNAc moiety is observed to be perpendicular to the UDP moiety and this conformation is stabilized by several hydrogen bonds, interaction with the pyrophosphate of UDP and hydrophobic interactions (Fig. 4 ▸ *b* and Supplementary Fig. S4*b* ). The C3 hydroxyl group of GlcNAc moiety is stabilized by Glu406 and the backbone amides of Gly407–Ser409, and the C6 hydroxyl group establishes a hydrogen bond to His246 of the ABD (Fig. 4 ▸ *b*). The hydroxyl groups of C4 and the N atom of an *N*-acetyl group form hydrogen bonds of 2.76 and 2.79 Å, respectively, to the pyrophos­phate of UDP (Fig. 4 ▸ *b*, inset). These interactions facilitate exposure of the anomeric sugar carbon (C1) to nucleophilic attack by an acceptor (Fig. 4 ▸ *b*). Therefore, the SdgB_quaternary_ structure presents a snapshot of the intermediate state after UDP-GlcNAc hydrolysis by SdgB on the pathway to the following glycosyl-transfer step onto the SD-repeat protein.

**Figure 5:**
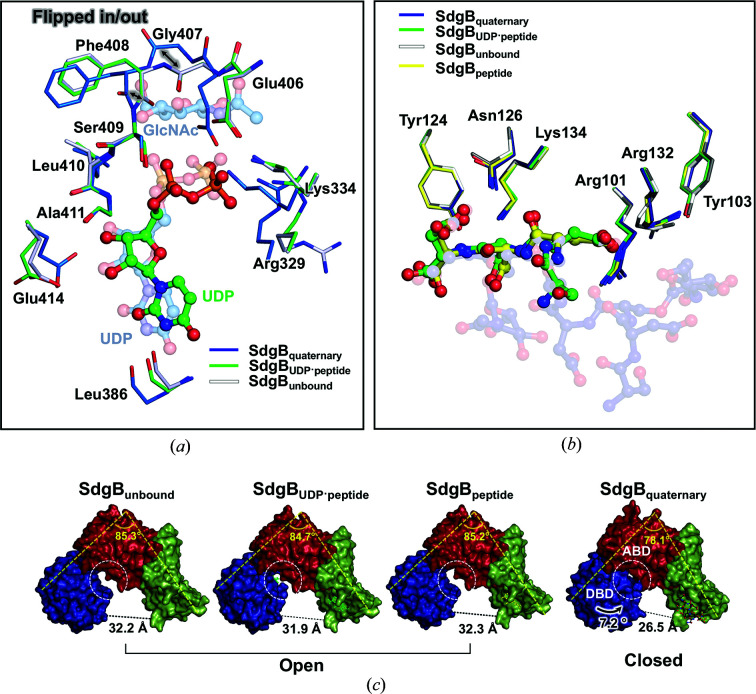
Structural comparison among the complexes of SdgB. (*a*, *b*) All ligands and the key binding residues observed in the UDP-GlcNAc (*a*) and SD-repeat peptide (*b*) binding sites of SdgB complexes were superimposed for comparison. The ligands of SdgB_quaternary_ are transparent. (*c*) Surface views of monomers of SdgB_unbound_ and complexes. The surface views are colored depending on domains as in Fig. 2 ▸(*a*). Yellow dotted and black dotted lines indicate the angles and distances. White dotted circles between the DBD and ABD indicate the open and closed conformations of the active-site cleft.

Surprisingly, the SdgB_quaternary_ structure reveals clear electron density for the GlcNAcylated SD-repeat peptide (9-mer), evidently as a product of catalysis in the crystal. The peptide is located in the positive groove inside the inserted (or dimerization) domain of SdgB, rather than in the ABD (Fig. 4 ▸ *a*). This result suggests that SD-repeat acceptor products can bind to the dimerization domain after being glycosylated by SdgB. Furthermore, electron density for the intact 3-mer (DSD) or 5-mer (DSDSD) SD-repeat peptides was found in this same area in the SdgB–peptide complexes described later and is illustrated in Fig. 5 ▸(*b*). It is likely that the SD-repeat acceptor substrates are first loaded into the positive groove of the dimerization domain before being glycosylated at the ABD. In sum, the dimerization domain of SdgB might serve as a binding platform for the SD-repeat acceptor substrates during the glycosylation process.

Despite the cocrystallization with the 9-mer peptide (^1^DSDSDSDS^9^D) as an acceptor substrate, the first aspartate (^1^Asp) could not be refined due to a lack of electron density induced by the flexibility in the SdgB_quaternary_ structure. In contrast to the expectation of full glycosylation, among the four serine residues of the 9-mer peptide, GlcNAcylations are found at two serine residues, ^4^Ser and ^8^Ser, but could not be confirmed at ^2^Ser and ^6^Ser (Fig. 4 ▸ *c*). The GlcNAcylated SD-repeat peptide product forms a partial 3_10_-helix (inset in Fig. 4 ▸ *a* and Supplementary Fig. S5) and makes extensive hydrophilic interactions with residues from the dimerization domain of SdgB. That is, the side chains of the mostly long charged residues Arg101, Tyr124, Asn126, Arg132, Lys134 and Arg137 interact with the side chains of Asp residues and the backbone carbonyl groups of the SD-repeat peptide via hydrogen bonds and salt bridges, which are especially concentrated at ^7^Asp and ^9^Asp (Fig. 4 ▸ *c*). Strong anchoring of the two Asp residues onto the shallow, positive groove (inset in Fig. 4*a*), which is mainly lined with Arg101, Arg132, Lys134 and Arg137, seems to facilitate the formation of a 3_10_-helix and further interactions of the backbone and the sugar moieties of the glycosylated SD-repeat peptide with the region. Aliphatic regions of the SD-repeat peptide are stabilized by the side-chain benzene rings of Phe108, Tyr111 and Phe128 (Supplementary Fig. S4*c* ). In addition, Tyr103 and Arg132 form hydrophilic interactions with the first *O*-GlcNAc moiety attached to ^4^Ser, suggesting that these residues may play a crucial role in the recognition of a glycosylated product (Fig. 4 ▸ *c*). The second *O*-GlcNAc moiety appended to ^8^Ser is stabilized by hydrophobic interactions with Ser97, Asp99 and Tyr111 (Supplementary Fig. S4*c* ).

### 3.5. Structural comparison of multiple SdgB–ligand complexes   

In addition to the SdgB_quaternary_ structure, structures of the ternary complex (SdgB_UDP–peptide_), which possesses UDP in the donor-binding site and the 5-mer peptide (DSDSD) in the dimerization domain, of the binary complex (SdgB_peptide_) complexed with the 3-mer peptide (DSD) and of the phosphate ion-bound form (SdgB_phosphate_) were determined. When SdgB_quaternary_ is superimposed on SdgB_UDP–peptide_ and SdgB_peptide_, modest changes in the donor substrate-binding positions could be observed (Fig. 2 ▸ *d*). The residues around the UDP of SdgB_UDP–peptide_ have similar conformations as in SdgB_unbound_, implying that SdgB_UDP–peptide_ is an inactive SdgB form (Fig. 5 ▸ *a*). The UDP moiety does not show interactions with Arg329, Lys334 and Glu414, which are key residues interacting with UDP in SdgB_quaternary_ that are conserved for the catalysis of glycosyltransferases (Shi *et al.*, 2014 ▸; Sobhanifar *et al.*, 2015 ▸; Hu *et al.*, 2003 ▸; Guerin *et al.*, 2007 ▸; Fig. 5 ▸ *a*). In the absence of the GlcNAc moiety in SdgB_UDP–peptide_, the carbonyl O atoms of Gly407 and Phe408 on the β21–α12 loop show the opposite arrangement to that in SdgB_quaternary_, which induces the major conformational change in the UDP-GlcNAc binding site (Fig. 5 ▸ *a*). In other words, the structural comparisons (Fig. 5 ▸ *a*) show that the presence of the GlcNAc moiety in the SdgB_quaternary_ structure induces flipping of the Gly407–Phe408 and Phe408–Ser409 peptide bonds so that the amide NH groups of these two peptides point inwards to make hydrogen bonds with GlcNAc, whereas in all other structures the carbonyl groups point inwards and the amides point outwards .

No electron density was visible for the first aspartate of the 5-mer DSDSD peptide in the SdgB_UDP–peptide_ structure, which only gave a refined model of the SDSD part (Supplementary Fig. S6). The 5-mer peptide bound in the SdgB_UDP–peptide_ and the 3-mer peptide (DSD) in the SdgB_peptide_ structure reveal a similar conformation to that of the glycosylated 9-mer peptide of SdgB_quaternary_ and are well matched at the positions from ^6^Ser to ^9^Asp (or ^7^Asp–^9^Asp) of the 9-mer peptide in SdgB_quaternary_, respectively. Also, the interacting residues do not show significant alterations across SdgB_quaternary_, SdgB_peptide_, SdgB_UDP–peptide_ and SdgB_unbound_ (Fig. 5 ▸ *b*). This confirms that the ^7^Asp and ^9^Asp residues and their interacting residues contribute greatly to the docking of the peptide to SdgB. Taken together, structural comparisons of the multiple states enhance the understanding of the recognition of donor and acceptor substrates.

### 3.6. SdgB structures with open and closed conformations   

The SdgB structures determined in this study reveal two conformational variations in its overall V-shaped monomer, depending on the types of the ligands bound (Figs. 2 ▸ *c* and 5 ▸ *c*). When comparing the per-residue main-chain r.m.s.d. over residues 1–496 between them, only the SdgB_quaternary_ structure displays large deviations in the DBD region compared with other structures (Fig. 2 ▸ *c*). SdgB_unbound_ has an open conformation, revealing that the active-site cleft between the DBD and ABD is open (Fig. 5 ▸ *c*). Despite the binding of UDP and/or the SD-repeat peptide, the SdgB_UDP–peptide_ and SdgB_peptide_ structures also have the open state shown by SdgB_unbound_ (Fig. 5 ▸ *c*). In contrast, the DBD of SdgB_quaternary_ rotates toward the ABD by 7.2° upon the binding of both UDP and GlcNAc, and the distance between the DBD and dimerization domain is shortened by 5.7 Å compared with that in SdgB_unbound_, resulting in a transition to the closed conformation (Fig. 5 ▸ *c*). These results suggest that the open to closed transition is induced only when both GlcNAc and UDP are present, presumably to reach the acceptor substrate for the subsequent glycosyl transfer.

**Figure 6:**
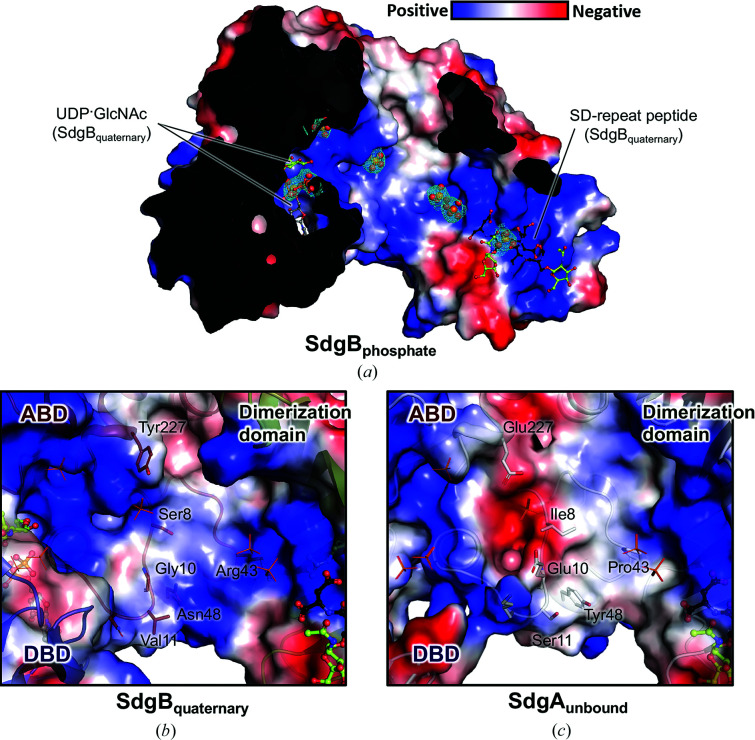
The putative binding paths for the negatively charged SDR substrates in the SdgB and SdgA structures. (*a*) The binding mode of multiple phosphate ions in the SdgB_phosphate_ structure. SdgB_phosphate_ is superimposed onto SdgB_quaternary_ and the phosphate ions are shown as sticks and spheres with a 2*mF*_o_ − *DF*_c_ electron-density map contoured at 1.0σ as a cyan mesh. The electrostatic surface model is presented using SdgB_quaternary_. (*b*, *c*) Charged surface views of the ABD of SdgB_quarternary_ (*b*) and SdgA_unbound_ (*c*). The representative residues constituting the surface are labeled. The SD-repeat peptide and phosphates complexed in SdgB_quaternary_ and SdgB_phosphate_, respectively, which are superimposed into SdgA_unbound_, are presented in stick models for comparison.

No electron density for an acceptor substrate bound to the ABD of SdgB was found in either the unmodified or modified SD-repeat peptide-bound complex structures. Instead, the complexes of SdgB showed the unique binding of the acceptor to the dimerization domain either as a substrate or product. Strikingly, our SdgB structures reveal the positively charged surface of SdgB extending from the active site to the dimerization domain (Fig. 4 ▸ *a*), leading us to speculate that natural SDR regions typically consisting of ∼300 amino acids (*i.e.* the SD repeat domain of ClfA) bind along this positively charged tract. In support of this speculation, the SdgB–phosphate complex (SdgB_phosphate_; PDB entry 7vfo) showed a series of seven phosphate ions lined up along the positively charged surface of SdgB (Fig. 6 ▸ *a*). Interestingly, when these phosphate ions are superimposed on SdgB_quaternary_, two of the phosphate ions overlap well with the pyrophosphate of UDP and one of the ions interacting with Lys134 shows a similar binding mode to the carbonyl group of ^8^Ser of SdgB_quaternary_ (Fig. 6 ▸ *a*). The good agreement of the phosphate ions with the substrates highlights that the remaining four phosphate ions spreading from the catalytic site to the dimerization domain may be a putative path for the binding of the long SD-repeat region found in the native SDR proteins. Since the 3–9-mer SD-repeat peptides complexed in the SdgB structures repeatedly show interactions of the peptides with the positive groove inside the dimerization domain, the conserved groove could be the first contact point for the SDR region. Subsequently, it is proposed that serine residues on the SD-repeat substrates might be glycosylated one after another in an ordered manner since they are consecutively lined up across the long positive tract from the dimerization domain to the active site. Collectively, the SdgB structures of the multiple states in this study provide insight into where the docking of the SD-repeat region onto SdgB commences and how those heavy modifications of numerous Ser sites clustered in the SDR domain can occur.

### 3.7. Comparison of the SdgB and SdgA structures   

Interestingly, the key residues that recognize the *O*-GlcNAc moieties of the glycosylated SD-repeat peptide in SdgB_quaternary_, as well as the residues interacting with the SD-peptide main chain, are strictly conserved both sequentially and structurally in the dimerization domain of SdgA (Supplementary Fig. S7), suggesting that SdgA might recognize a mono-glycosylated SD-repeat substrate in the same way as SdgB does. Also, the UDP-GlcNAc-sensing residues observed in the DBD of SdgB_quaternary_ are completely conserved in the SdgA structure. The overall structure of SdgA_unbound_ also has a conformation similar to the open state of SdgB_unbound_, even though the inner groove of SdgA_unbound_ appears to be wider and longer than that of SdgB_unbound_ (Supplementary Fig. S8). However, the ABD of SdgA possesses different residue propensities on its surface, especially along the long tract from the dimerization domain to the active site. The surface representation of the ABD in SdgA_unbound_ revealed an acidic and hydrophobic charge distribution compared with the SdgB_quaternary_ structure, indicating that the long positive track connected to the active-site cleft in the SdgB structure is no longer connected in the SdgA structure (Figs. 6 ▸ *b* and 6 ▸ *c*). In particular, the SdgB residues towards the putative acceptor substrate-binding site in the ABD, such as Ser8, Gly10, Val11, Asn48 and Tyr227, are substituted with Ile8, Glu10, Ser11, Tyr48 and Glu227, respectively, in SdgA. Additionally, not all residues located in the long positive tract in the SdgB dimerization domain and interacting with phosphate ions in SdgB_phosphate_ are conserved in SdgA: Tyr265, Arg224, Arg150, Arg137, Asn126 and Lys134 are conserved, whereas Tyr227, Asn7 and Arg43 are changed to Glu227, Thr7 and Pro43, respectively, in SdgA (Supplementary Fig. S7). These changes seem to make the substrate-binding platform in SdgA less favorable for the employment of the acidic SD-repeat substrate compared with that in SdgB. This might explain why SdgA was not able to modify short SDR peptides (3-mer and 5-mer SD peptides) in our *in vitro* glycosylation assay in contrast to SdgB (Figs. 1 ▸ *g* and 1*h*).

## 4. Discussion   

Host innate immune systems are the first line of defense against invading pathogens. Many invasive bacteria try to avoid detection and elimination by host immune reactions, and thus they have evolved diverse strategies that counteract the host defense machinery (Akira *et al.*, 2006 ▸). One method of bacterial immune evasion is glycosylation, a common post-translational modification in diverse organisms (Lin *et al.*, 2020 ▸). Protein glycosylation in bacteria promotes them to attack host proteins and enhances their virulence while acting as a barrier to protect them from the host immune responses (Thomer *et al.*, 2014 ▸; Hazenbos *et al.*, 2013 ▸). The glycosyltransferases SdgB and SdgA from *S. aureus* play a crucial role in staphylococcal coagulation and adhesion through the *O*-GlcNAcylation of the SDR region of virulence factors. The structural basis of GlcNAc transfer by SdgB and SdgA may provide a further understanding of bacterial mechanisms to avoid the host innate immune response.

In this study, we determined crystal structures of SdgB and SdgA at the atomic level, together with unique snapshots of various protein complexes each containing donor and/or acceptor ligands. As expected, SdgB and SdgA possess the GT-B fold consisting of two β/α/β Rossmann-like domains, a common fold among glycosyltransferases (Chang *et al.*, 2011 ▸; Gloster, 2014 ▸), and the distinct domains form donor and acceptor sites at the resulting cleft. Apart from these two domains, a conserved domain (DUF1975) was inserted into the acceptor-binding domain (Wu & Wu, 2011 ▸), which contributed to dimerization in the crystal structures of SdgB and SdgA. Further verification using SEC-MALS, SV-AUC and SE-AUC showed that SdgB and SdgA may act in a dimeric form in a physiological environment, but that SdgB has a tenfold stronger tendency to achieve the dimeric form than SdgA. Since the oligomeric state of *O*-GlcNAcyltransferases is importantly associated with their catalytic activity (Sobhanifar *et al.*, 2015 ▸; Chen *et al.*, 2016 ▸), this may be one of the factors contributing to the catalytic difference between SdgB and SdgA in the sequential transfer of the *O*-GlcNAc moiety. Moreover, as it was shown that SdgB and SdgA, which are simultaneously expressed with the SDR protein in the same operon, can interact with each other in solution, we suggest that the inserted domains of SdgB and SdgA can be combined in various forms to achieve homo- and hetero-dimerization.

Here, we have described diverse complexes of SdgB with ligands. Among them, SdgB_quaternary_ revealed the unique quaternary binding mode of SdgB, UDP, cleaved GlcNAc and a GlcNAcylated SD-repeat peptide, thereby providing a snapshot of the intermediate state after UDP-GlcNAc has been cleaved by SdgB on the pathway to the subsequent glycosyl-transfer step onto the SD-repeat protein. Comparison of the multiple SdgB–ligand complexes revealed that the loop consisting of Glu406–Ala411 plays a crucial role in the conformation of the donor-binding site. In particular, flips of the Gly407–Phe408 and Phe408–Ser409 peptide bonds not only have a significant effect on the interaction of the β-phosphate of UDP with the conserved catalytic residues Arg329 and Lys334 that are involved in the hydrolysis of UDP-GlcNAc, but also importantly contribute to the accommodation of and interactions with the cleaved GlcNAc. In addition, this structural change of the loop in the donor-binding site appears to induce an open-to-closed transition of SdgB. In fact, this transition has been demonstrated in diverse structural and biochemical studies of the GT-B superfamily, and it was proposed that such a molecular motion would be crucial for accommodating larger substrates, such as dis­ordered regions in folded proteins (Buschiazzo *et al.*, 2004 ▸; Guerin *et al.*, 2009 ▸; Sobhanifar *et al.*, 2015 ▸; Chen *et al.*, 2016 ▸; Janetzko & Walker, 2014 ▸).

Remarkably, SD-repeat peptide-complexed structures displayed redundant binding in the positive groove inside the dimerization domain of SdgB in both GlcNAcylated and unmodified states. To the best of our knowledge, this unique binding mode of modified or unmodified acceptor substrates in the DUF1975 dimerization domain has not previously been reported. It means that this spot inside the dimerization domain plays a role as a platform to which the SD-repeat region can be preferentially attached before and after the reaction, whereas the outside of the domain contributes to the oligomerization of SdgB. Furthermore, details of the binding mode between the positive groove and SD-repeat peptides provides insight into how the groove recognizes the SD-repeat region.

Through structural comparison between the complexes of SdgB illustrated in this study, we concluded that the presence of GlcNAc in the donor-binding domain induces an open-to-closed transition of SdgB to facilitate the approach of the acceptor towards the active site. A surface electrostatics analysis of SdgB showed a prominent positive groove extending along the surface from the active site at the N-terminal ABD to the dimerization domain, which may anchor the negatively charged SDR substrate during or after glycosylation. In the study of TarM (Sobhanifar *et al.*, 2015 ▸), this positive groove with sulfate ions was observed as a putative binding path for its substrate. Correspondingly, SdgB_phosphate_ illustrates an ordered binding of seven phosphate ions that lie along the positively extended groove. Therefore, we propose this phosphate-binding positive-charged tract as a putative binding path for the extended negative SDR acceptor of substrate proteins. Collectively, the structural studies of multiple SdgB complexes reveal that SdgB adopts diverse forms during the glycosyl-transfer process: homodimerization, heterodimerization with SdgA and a conformational change from open to closed upon UDP-GlcNAc binding.

Finally, SdgA shares considerably high similarity with SdgB sequentially and structurally, and in particular the key residues recognizing UDP-GlcNAc in the DBD or the glycosylated SD-repeat peptide in the dimerization domain are strictly conserved, implying that the structures of the diverse complexes of SdgB would be reminiscent of the ligand-interacting modes of SdgA. Mass-spectrometric analysis showed that SdgB alone could append the GlcNAc moiety to the serine residue of a short SDR peptide, whereas SdgA alone could not modify it. Additionally, the second GlcNAcylation by SdgA could not be detected when both SdgB and SdgA were added to the reaction. As it has been shown that SdgB and SdgA exhibit their activities in the sequential modification of a long SDR peptide (Hazenbos *et al.*, 2013 ▸; Thomer *et al.*, 2014 ▸), it is suggested that SdgA could not target the short GlcNAcylated peptide for further modification. Considering the sequential GlcNAcylation of SDR domains by these two enzymes, we expected noticeable structural differences between the active sites of the two enzymes. Notably, when compared with SdgB, the long positive tract leading to the positive groove of the dimerization domain seen in SdgB is not well formed on the surface of the ABD in SdgA, but rather shows a more hydrophobic and acidic charge distribution. The interruption of the long positive tract due to different charge propensities on the surface of the ABD in SdgA may be the reason why SdgA failed to perform additional glycosylation of the short peptide, suggesting that SdgA preferentially accommodates a long SD-repeat molecule that could be docked in both the active-site cleft and the positive groove of the dimerization domain.

In conclusion, here we have reported the crystal structures of SdgB and SdgA, which are responsible for the processive *O*-GlcNAcylation of the alternate serine residues of the SDR domain of pathogenic proteins from *S. aureus*. Our complexes of SdgB with SDR peptides show that the insertion domain DUF1975 directly recognizes its acceptor substrate as well as promotes dimerization. Together with biophysical and biochemical analyses, the diverse snapshots of the five complexes of SdgB provide the molecular basis of the catalytic mechanism. Therefore, our findings reveal valuable insights into the molecular mechanisms of SdgB and SdgA, and will provide a novel strategy for the development of alternative therapeutic agents against staphylococcal infections.

## 5. Related literature   

The following references are cited in the supporting information for this article: Gouet *et al.* (1999 ▸) and Tina *et al.* (2007 ▸).

## Supplementary Material

PDB reference: SdgB_unbound_ (ligand-free), 7vfk

PDB reference: SdgA_unbound_ (ligand-free), 7ec2

PDB reference: SdgB_quaternary_ (SdgB–UDP–GlcNAc–diGlcNAcylated peptide complex), 7vfl

PDB reference: SdgB_UDP–peptide_ (SdgB–UDP–5-mer complex), 7vfm

PDB reference: SdgB_peptide_ (SdgB–3-mer complex), 7vfn

PDB reference: SdgB_phosphate_ (SdgB–phosphate complex), 7vfo

Supplementary Figures. DOI: 10.1107/S2059798321010068/jb5030sup1.pdf

## Figures and Tables

**Table 1:** Crystallographic data-collection and refinement statistics Values in parentheses are for the highest resolution shell.

Data set	1	2	3	4	5	6	7
Structure	SdgB_SeMet_	SdgB_unbound_	SdgA_unbound_	SdgB_quaternary_	SdgB_UDP–peptide_	SdgB_peptide_	SdgB_phosphate_
Data collection							
Beam source	PF-17A	PLS-7A	PLS-5C	PF-1A	PLS-7A	PLS-7A	NE-3A
X-ray wavelength (Å)	0.9791	0.9793	0.9796	1.1000	0.9794	0.9793	1.0000
Space group	*P*2_1_2_1_2_1_	*P*2_1_	*C*2	*P*2_1_2_1_2_1_	*P*2_1_	*P*2_1_	*P*3_2_21
*a*, *b*, *c* (Å)	67.9, 111.9, 190.7	49.9, 206.3, 66.3	177.9, 61.4, 111.6	70.2, 130.6, 189.9	42.7, 206.3, 66.1	43.1, 206.2, 66.4	172.3, 172.3, 106.2
α, β, γ (°)	90.0, 90.0, 90.0	90.0, 105.1, 90.0	90.0, 122.5, 90.0	90.0, 90.0, 90.0	90.0, 105.0, 90.0	90.0, 105.3, 90.0	90.0, 90.0, 120.0
Resolution range (Å)	50.00–3.40 (3.46–3.40)	30.00–1.84 (1.88–1.84)	50.00–2.80 (2.85–2.80)	50.00–2.50 (2.54–2.50)	50.00–2.28 (2.32–2.28)	30.00–1.90 (1.93–1.90)	50.00–3.20 (3.26–3.20)
Total/unique reflections	418964/20668	358372/93867	98757/25354	330271/60708	231254/49800	357664/87189	220937/26949
Completeness (%)	100.0 (100.0)	98.4 (99.5)	99.4 (99.8)	96.7 (95.1)	99.6 (98.5)	99.3 (100.0)	89.0 (99.7)
CC_1/2_ †	0.994 (0.979)	0.996 (0.708)	0.938 (0.710)	0.934 (0.776)	0.995 (0.806)	0.992 (0.776)	0.973 (0.916)
Multiplicity	20.3 (20.8)	3.8 (3.8)	3.9 (4.0)	5.4 (5.4)	4.6 (3.9)	4.1 (4.1)	8.2 (9.1)
〈*I*/σ(*I*)〉	28.0 (9.81)	26.3 (1.92)	18.6 (2.25)	12.9 (1.76)	27.5 (2.62)	26.0 (2.29)	16.9 (4.18)
*R*_merge_ ‡	0.178 (0.544)	0.062 (0.741)	0.085 (0.603)	0.107 (0.715)	0.084 (0.628)	0.082 (0.775)	0.154 (0.502)
Refinement							
PDB code		7vfk	7ec2	7vfl	7vfm	7vfn	7vfo
*R*_work_/*R*_free_ §		0.217/0.258	0.236/0.289	0.182/0.240	0.213/0.274	0.220/0.260	0.197/0.262
No. of atoms							
Protein		8228	7655	8334	8176	8183	8254
Water		223	32	24	17	195	4
Ligand		52	—	221	94	22	55
Average *B* factor (Å^2^)							
Protein		40.1	67.2	41.0	53.6	40.4	59.3
Water		35.1	47.3	29.2	38.7	34.8	22.4
Ligand		51.8	—	50.0	77.2	51.2	64.1
R.m.s. deviations from ideal geometry							
Bond lengths (Å)		0.008	0.005	0.016	0.018	0.013	0.011
Bond angles (°)		0.954	1.379	1.264	1.329	1.329	1.246
Ramachandran plot (%)							
Most favorable		96.93	92.36	96.82	95.58	96.07	94.51
Allowed		3.07	7.64	2.88	4.42	3.93	5.49
Disallowed		0.00	0.00	0.30	0.00	0.00	0.00

† CC_1/2_ is described in Karplus & Diederichs (2012 ▸).

‡ *R*_merge_ = \textstyle \sum_{hkl}\sum_{i}|I_{i}(hkl)- \langle I(hkl)\rangle|/\textstyle \sum_{hkl}\sum_{i}I_{i}(hkl), where *I*(*hkl*) is the intensity of reflection *hkl*, \textstyle \sum_{hkl} is the sum over all reflections and \textstyle \sum_{i} is the sum over *i* measurements of reflection *hkl*.

§ *R* =\textstyle \sum_{hkl}\big ||F_{\rm obs}|-|F_{\rm calc}|\big |/ \textstyle \sum_{hkl}|F_{\rm obs}|, where *R*_free_ is calculated for a randomly chosen 5% of reflections which were not used for structure refinement and *R*_work_ is calculated for the remaining reflections.
