# Supplementary material for: Structural basis for SdgB- and SdgA-mediated glycosylation of staphylococcal adhesive proteins
Source: Acta Crystallogr D Struct Biol. 2021 Oct 20;77(Pt 11):1460–74. doi: 10.1107/S2059798321010068 (PMC8561734; doi:10.1107/S2059798321010068)
Supplement: Supplementary file 1 [file d-77-01460-sup1.pdf]

STRUCTURAL
BIOLOGY

Volume 77 (2021)

Supporting information for article:

Structural basis for SdgB- and SdgA-mediated glycosylation of staphylococcal adhesive proteins

Dong-Gyun Kim, Inwha Baek, Yeon Lee, Hyerry Kim, Jin Young Kim, Geul Bang, Sunghwan Kim, Hye Jin Yoon, Byung Woo Han, Se Won Suh and Hyoun Sook Kim

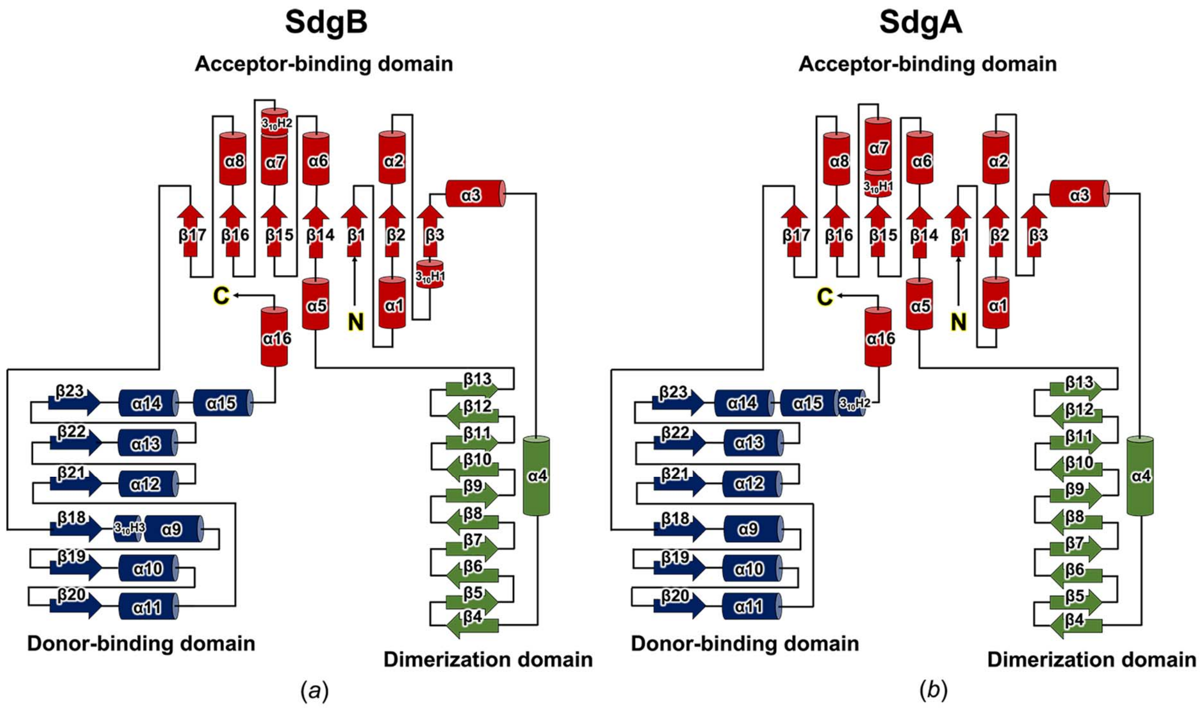

Figure S1 Topology diagrams of SdgB and SdgA. Helices and strands are shown as a cylindrical and ribbon diagram, respectively. Loops are shown in black line. Red, green, and blue diagrams indicate acceptor-binding domain, dimerization domain, and donor-binding domain, respectively. Secondary structures were calculated by Espript 3 (Gouet *et al.*, 1999) based on the coordinates of $SdgB_{quaternary}$ and $SdgA_{unbound}$.

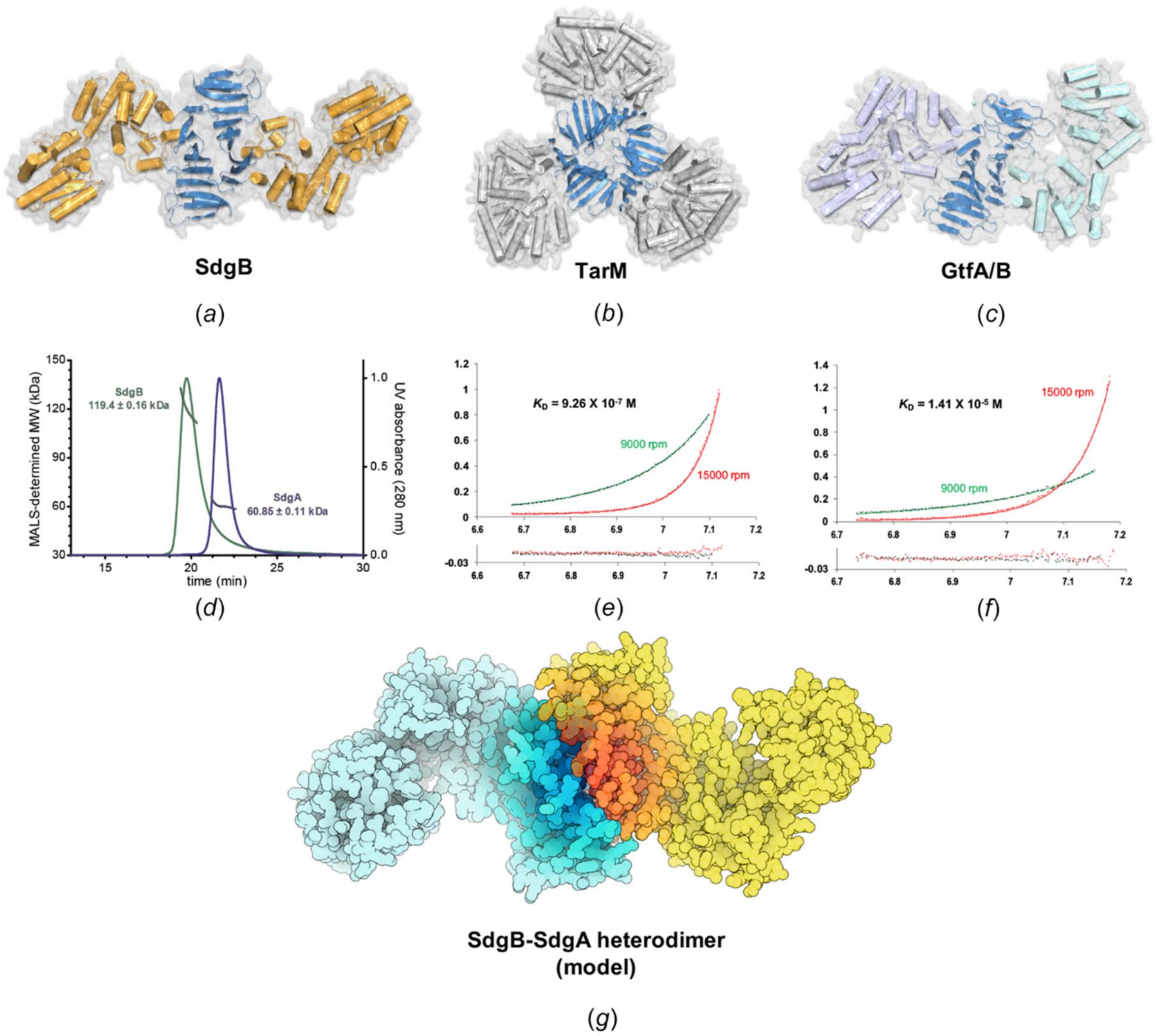

Figure S2 Oligomeric state analysis of SdgB and SdgA. (*a*–*c*) Oligomerization of SdgB (*c*) and its homologs, TarM (*b*) (PDB entry: 4X6L) and GtfA/B (*c*) (PDB entry: 5E9T). The key domains (DUF1975) contributing to oligomerization are marked as blue. (*d*) Assembly analysis of SdgB and SdgA by SEC-MALS. In solution, the SdgB and SdgA proteins of 0.22 mg and 0.23 mg, respectively (theoretical M.W. of monomers including an affinity tag = 59.5 and 58.3 kDa, respectively) exist as dimer and monomer of 119.4 and 60.85 kDa, respectively. (*e*, *f*) Equilibrium sedimentation data for SdgB (*e*) and SdgA (*f*) at ultracentrifugal speeds of 9,000 and 15,000 rpm are shown in upper panels. The sedimentation data set was globally fit to monomer-dimer self-association model. The residual differences (Abs_{fit}-Abs_{obs}) (lower panels) indicate a good quality fit in agreement with monomer-dimer equilibrium. (*g*) Surface representation of the model structure of the SdgB-SdgA heterodimer. SdgB and SdgA are gradiently colored cyan to blue and yellow to brown, respectively, as the proximity of the interface between them.

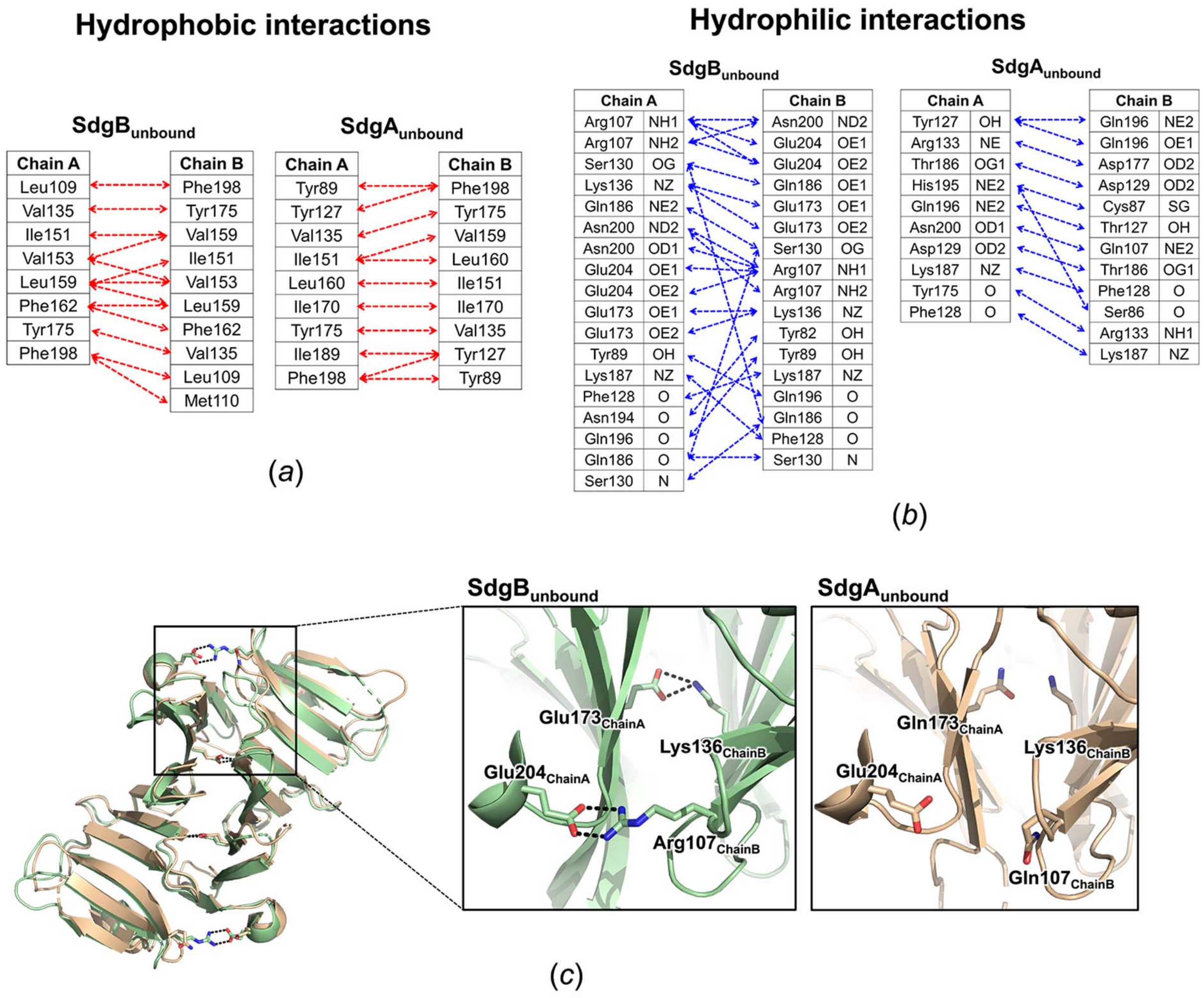

Figure S3 Residual interactions in the dimeric interfaces of SdgB and SdgA. Hydrophobic (*a*) and hydrophilic (*b*) interactions contributing to the dimeric conformation of SdgB and SdgA. (*c*) Comparison of the residues forming salt bridges in SdgB and SdgA. The dimerization domains of SdgB (green) and SdgA (wheat) are superimposed. The right-enlarged views show the residues forming the salt bridges in SdgB and the corresponding residues in SdgA. All interactions were calculated by the Protein Interactions Calculator (PIC) server (Tina *et al.*, 2007)

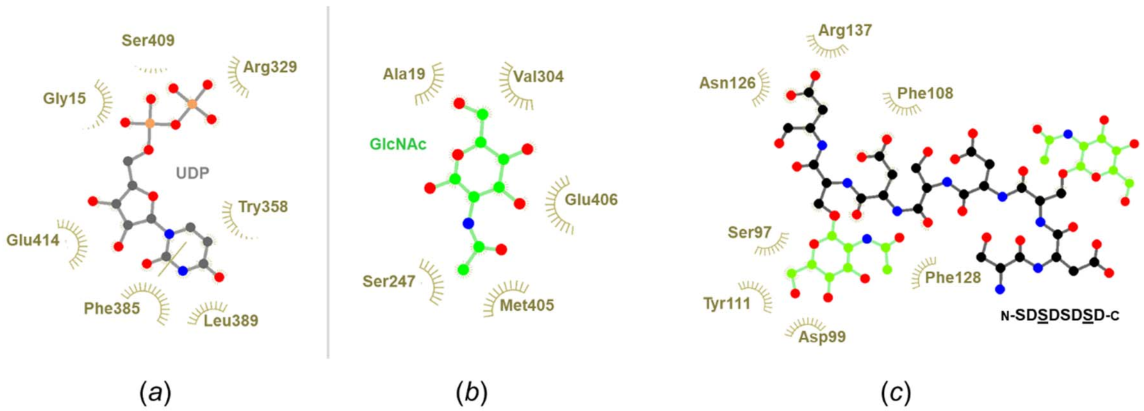

Figure S4 Schematic representation of hydrophobic interactions of SdgB with the UDP (*a*), GlcNAc (*b*), and GlcNAcylated SD-repeat peptide (*c*) using LIGPLOT diagram. The interactions and the contributing residues are demarcated by light-yellow spoked arcs.

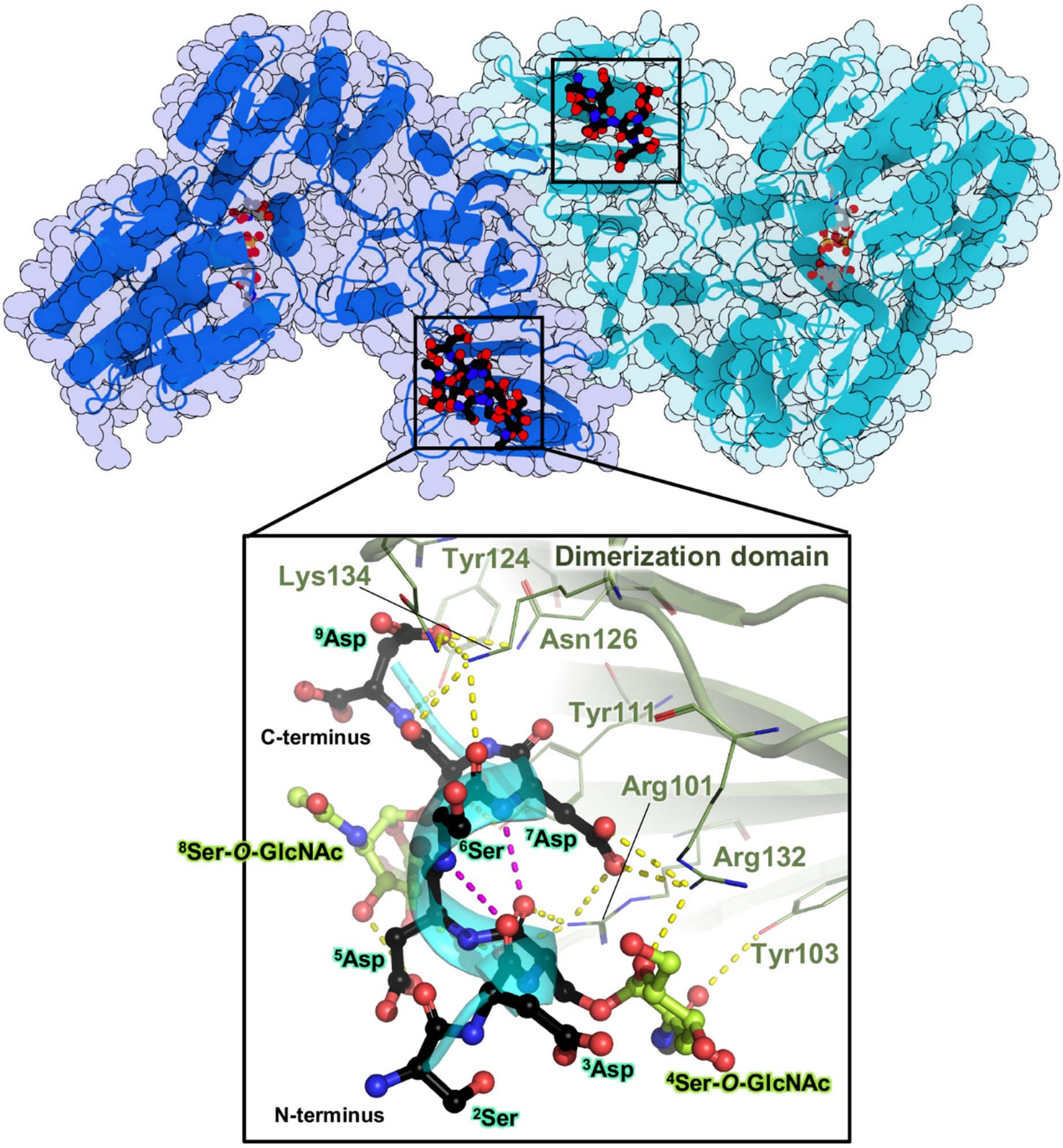

Figure S5 The binding mode of glycosylated-SD peptide in the dimeric SdgB$_{quaternary}$ structure. The yellow or pink-dotted lines indicate inter- or intramolecular interactions, respectively. 310-helix of the peptide is presented as ribbon diagram colored as sky.

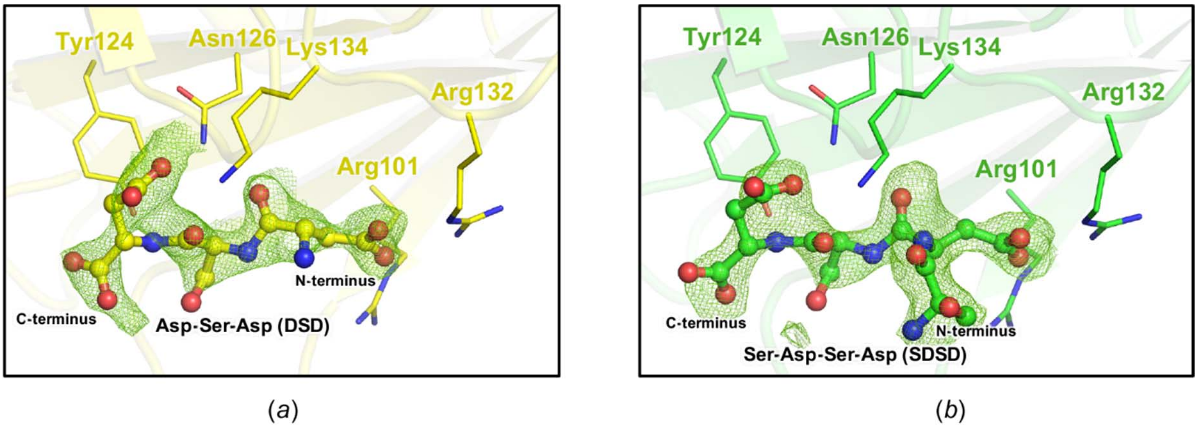

Figure S6 The omit maps of the respective peptides in $\text{SdgB}_{\text{peptide}}$ and $\text{SdgB}_{\text{UDP}\cdot\text{peptide}}$. $mF_o{-}DF_c$ electron density maps are presented at the contour of 2.0 σ (*a*) and 1.5 σ (*b*) with green meshes.

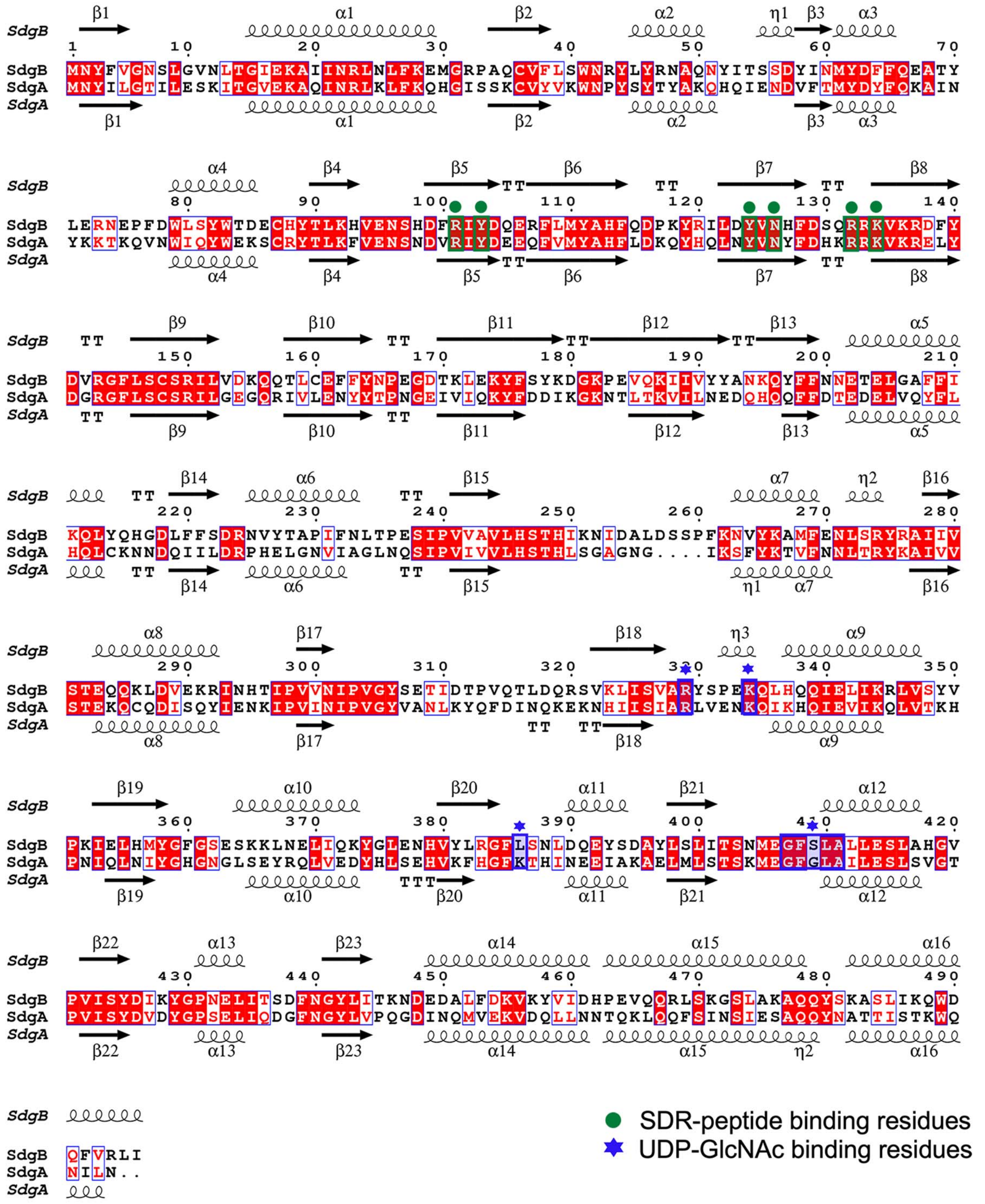

Figure S7 Structure-based sequence alignment of SdgB and SdgA. The β-strands (black arrow) and α-helices (black helix) are based on $SdgB_{unbound}$. The highly conserved amino acid residues are presented as white letters on a red background. Amino acid residues that have comparable chemical and physical properties are presented as red letters within blue frames. The green circles and blue-stars highlight the key residues for SDR-peptide binding or UDP-GlcNAc binding, respectively. The alignment was obtained using ESPript (Gouet *et al.*, 1999).

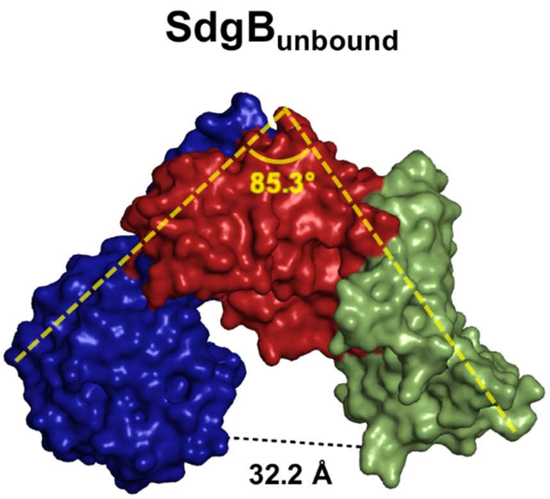

Figure S8 Surface views of monomers of $SdgB_{unbound}$ and $SdgA_{unbound}$. The surface views are colored as in Fig. 2(*a*). Yellow-dotted and black-dotted lines indicate the angles and distances.
